# γδ T cells as early sensors of tissue damage and mediators of secondary neurodegeneration

**DOI:** 10.3389/fncel.2014.00368

**Published:** 2014-11-05

**Authors:** Mathias Gelderblom, Priyadharshini Arunachalam, Tim Magnus

**Affiliations:** Department of Neurology, University Medical Center Hamburg-EppendorfHamburg, Germany

**Keywords:** γδ T cell, stroke, inflammation, IL-17, lymphocyte, brain, ischemia, neutrophils

## Abstract

Spontaneous or medically induced reperfusion occurs in up to 70% of patients within 24 h after cerebral ischemia. Reperfusion of ischemic brain tissue can augment the inflammatory response that causes additional injury. Recently, T cells have been shown to be an essential part of the post-ischemic tissue damage, and especially IL-17 secreting T cells have been implicated in the pathogenesis of a variety of inflammatory reactions in the brain. After stroke, it seems that the innate γδ T cells are the main IL-17 producing cells and that the γδ T cell activation constitutes an early and mainly damaging immune response in stroke. Effector mechanism of γδ T cell derived IL-17 in the ischemic brain include the induction of metalloproteinases, proinflammatory cytokines and neutrophil attracting chemokines, leading to a further amplification of the detrimental inflammatory response. In this review, we will give an overview on the concepts of γδ T cells and IL-17 in stroke pathophysiology and on their potential importance for human disease conditions.

## Introduction

Ischemic stroke is the primary reason for sustained disability and the third leading cause of death in the western world. In 85% of these patients, occlusion of an artery in the brain is the cause of stroke. Early restoration of blood flow (reperfusion) remains the treatment of choice for limiting brain injury following stroke. The reperfusion, which enhances the oxygen and glucose content in the tissue also increases an inflammatory response (Iadecola and Anrather, 2011). The idea that inflammation causes further brain injury is supported by a large number of reports that describe a reduction in infarct size and brain edema in animal models of stroke that receive blocking antibodies against specific cell adhesion molecules that mediate leukocyte recruitment (Yilmaz and Granger, 2008), anti-inflammatory treatment (Sharkey and Butcher, 1994), and immune deficient animals (Yilmaz et al., 2006; Hurn et al., 2007; Kleinschnitz et al., 2010; Gelderblom et al., 2012).

## αδ T cells and regulatory T cells in stroke

Compared to resident microglia, infiltrating macrophages and neutrophils, lymphocytes and NK cells infiltrate the ischemic hemisphere in small numbers. Nevertheless, T cells have a great impact on stroke outcome. The initial observation by Yilmaz et al. that lymphocyte deficient rag1^−/−^ mice are protected from stroke (Yilmaz et al., 2006) could be extended to mice with severe combined immunodeficiency lacking T cells and B cells (Hurn et al., 2007) and to CD4^+^ and CD8^+^ T cell-deficient animals (Yilmaz et al., 2006). Direct detrimental mechanisms elicited by αβ T cell in stroke pathophysiology include CD8^+^ T cell derived perforin mediated cytotoxicity (Liesz et al., 2011) and IL-21 secreted by CD4^+^ T cells (Clarkson et al., 2014).

The classical activation of αβ T cells requires several coincident signals: (1) engagement of the antigen receptor; (2) co-stimulatory receptors; (3) cytokine receptors such IL-2 receptor; a process requiring at least 3–5 d (Jensen et al., 2008). Multiple studies using antigen specific mucosal tolerization protocols against myelin antigens suggest the involvement of adaptive mechanism in stroke pathophysiology. Already in 1997 the group from Hallenbeck demonstrated that rodents tolerized with myelin peptides are protected from ischemic stroke (Becker et al., 1997). Mechanistically the protective effects could be attributed to IL-10 producing T cells (Frenkel et al., 2005) and transforming growth factor-β1 (Becker et al., 2003).

These classical concepts of T cell activation are challenged by the observation that detrimental T cell dependent effects following cerebral ischemia can be observed already 24 h post stroke, in an antigen independent fashion (Kleinschnitz et al., 2010). Similarly controversial is the role of regulatory T_regs_ and B cells in stroke. Liesz and colleagues showed that endogenous T_regs_ are protective in later stages following stroke when the lesions were small (Liesz et al., 2009) and that their beneficial functions depend on IL-10 (Liesz et al., 2013). However, a lot of the observed effects of T_regs_ cannot be attributed to concepts of adaptive immunity. For example, an early direct inhibitory effect of T_regs_ on the MMP9 production from neutrophils was a recently suggested mechanism (Li et al., 2013). In this model, transfer of regulatory T_regs_ conferred protective effects on the outcome already on day one after stroke even before T_regs_ infiltrated the ischemic brain. Protective effects could be attributed to program death-1 ligand 1 (PD-L1) dependent inhibition on MMP9 production in neutrophils in the peripheral circulation which then led to a consecutive protection of the blood brain barrier (Li et al., 2014). Further studies even challenged the overall concept of T_regs_ as endogenous protective immune cell population in stroke (Ren et al., 2011) and a recent study suggests that T_regs_ have an early detrimental role, by inducing dysfunction of the cerebral microcirculation (Kleinschnitz et al., 2013). While the data on T cell effects in particular T_regs_ in stroke is still controversial, it is clear that most of the important immunological effects are not following classical concepts of adaptive immunity, suggesting an innate like behavior of lymphocytes. In this line, atypical T cells such as γδ T cell and NK cells are likely to participate in the early orchestration of the inflammatory reaction. For NK cells it has been shown that neuronal cell death is mediated by IFN-γ- and Perforin-dependent pathways as early as 3 h post reperfusion (Gan et al., 2014). A lot more data exist on γδ T cell, which we will focus on in the following section.

## Biology of γδ T cells subpopulations

Like αβ T cells, γδ T cells develop in the thymus using the recombinase activated gene product (RAG) for the somatic rearrangement of V (variable), D (Diversity and J (joining) gene segments of the γ and δ chains of their T cell receptor (TcR) (reviewed in Raulet, 1989). Compared to αβ TcR, the sets of TcR detected on γδ T cells are limited. Many γδ subsets, primarily the ones populating certain tissues such as the epidermis, dermis, intestine, lungs and uterus are displaying an even higher limitation of their TcR diversity. These tissue-specific γδ T cell subsets show a biased use of certain TcR V gene segments. Since some of them express “invariant” TcRs with identical (canonical) junctional sequences, they are also named canonical γδ T cells. As reviewed by Vantourout and Hayday, the limited TcR diversity implies that these cells recognize either pathogen encoded antigens, that are likely to be encountered in specific tissues such as the epidermis, or self-encoded molecules that reflect a dysregulated state of that tissue (Vantourout and Hayday, 2013). Since these γδ T cell subsets can be rapidly activated without the requirement of prior clonal expansion they are also called “innate like” T cells. In contrast to canonical γδ T cells so-called non-canonical γδ T cells, which are characterized by an expression of more diverse γδ TcRs, are homing into secondary lymphoid tissues. Here they make up a minor fraction of rodent and human T cells after birth (in mice 1–4% of all T cells). In the context of immune responses non-canonical γδ T cells are capable to participate distant from their original site of residence, by trafficking to the site of inflammation in solid organs (reviewed by Korn and Petermann, 2012). Similar to αβ T cells, γδ T cells can be divided by their cytokine profile. Mouse γδ T cells which are developing from fetal liver progenitors undergo functional pre-programming, which leads to a subpopulation of IL-17 producing Scart-2^+^ and CCR6^+^ γδ T cells on one side and IFN-γ producing NK.1.1^+^ and CD27^+^ γδ T cells on the other side. Both subpopulations have an innate like phenotype, since they can be rapidly activated without prior clonal expansion (Vantourout and Hayday, 2013). γδ T cells fulfill important sentinel functions in the immune system. The ability of γδ T cells to recognize molecules that are rapidly displayed after stress without requiring extensive clonal expansion permits γδ T cells to participate in early stages of immune responses. In such scenarios γδ T cells act in parallel with cells of the innate immune system as sensors of dysregulation. γδ T cells may respond to classical signals of the adaptive immune system or to cytokine signals and either Toll-like receptor (TLR) or dectin stimuli in the absence of TcR ligation. Activation of the γδ TcR can occur through major histocompatibility complex (MHC)-related and unrelated TcR ligands, which are including foreign- and self-antigens. This allows γδ T cells to respond to infection and sterile tissue dysregulation such as ischemia. Beside TcR dependent mechanisms γδ T cell activation can be mediated through engagement of the activating natural killer receptors (NKRs) such as NK group 2 member D (NKD2D), by patter recognition receptors including TLRs (reviewed by Bonneville et al., 2010) and through cytokines such as IL-1β and/or IL-23 (Sutton et al., 2009). The constitutive expression of IL-23 and IL-1β receptors by γδ T cells assures this rapid responsiveness. Within hours upon activation and without prior expansion systemic γδ T cells can express high levels of effector cytokines, such as IFN-γ, IL-17, TNF-α and granzymes. In addition, γδ T cells are capable of producing numerous chemokines and regulatory factors including IL-13 and insulin-like-growth factor 1 (IGF-1), allowing them to interact with other immune cells, such as B cells and αβ T cells in the afferent phase of the immune response. Regarding the cellular interplay between γδ T cells and innate immune cells neutrophils play a central role. Once activated, γδ T cells can stimulate the release of potent chemoattractants for neutrophils. In this respect, γδ T cells were recently shown to be the primary sources of the neutrophil-attracting IL-17 in mouse models of infection (Shibata et al., 2007), hypersensitivity (Simonian et al., 2009) and autoimmunity (Roark et al., 2007). Often the activation of the innate immune system results in a feed back loop that increasingly stimulates γδ T cells.

## γδ T cells as sensors of tissue damage in stroke

**Figure 1:**
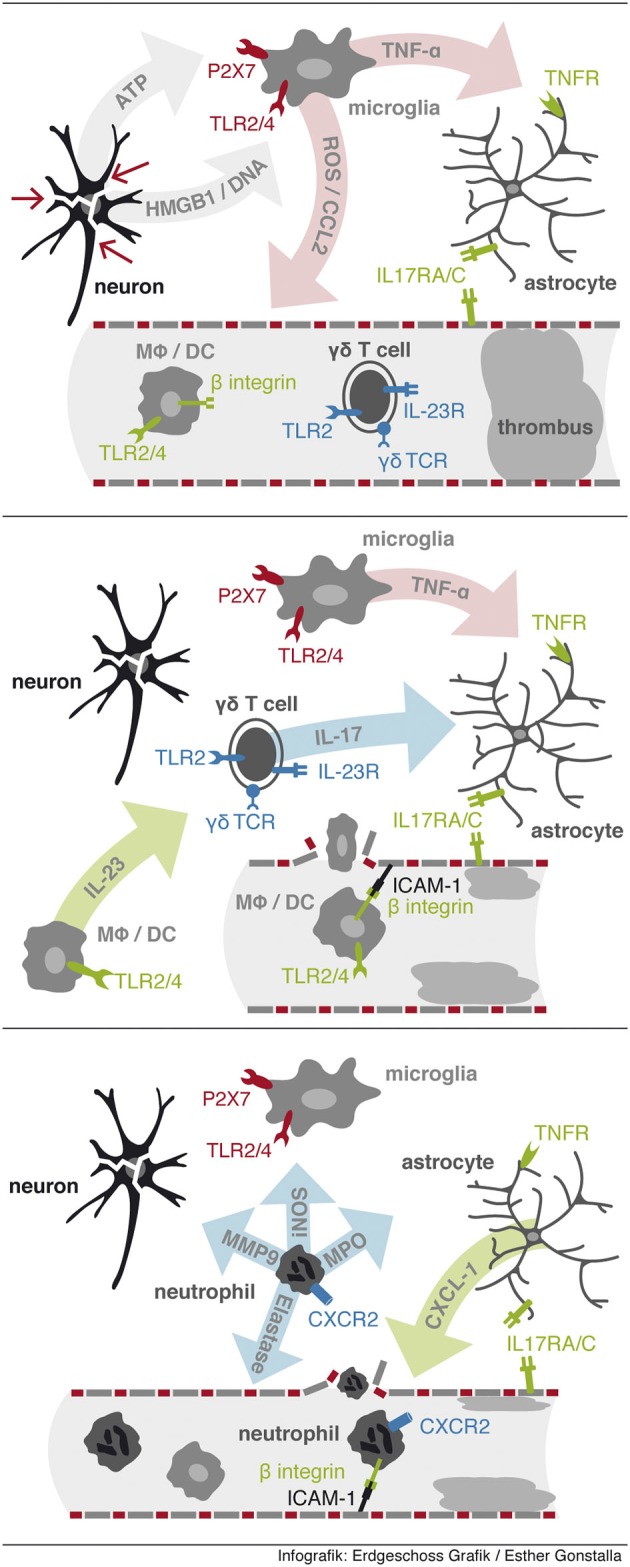
**Sequential events leading to neutrophil infiltration**. First, release from DAMPs from injured cells activates resident microglia via PPRs to release proinflammatory factors including TNF-α. Second, IL-23 activates γδ T cells to rapidly secrete IL-17 in the ischemic tissue. Third, neutrophil infiltration is initiated via IL-17 and TNF-α synergistically induced expression of CXCL-1 in astrocytes.

Stroke resembles classical features of a “sterile inflammation”, which is characterized by a inflammation in response to tissue disruption without the involvement of pathogenic microorganisms (See Figure 1; Chen and Nuñez, 2010). Sterile inflammation shares similar mechanisms with inflammation during infection. Receptors essential for sensing microorganisms are collectively called pattern recognition receptors (PPRs). PRRs sense conserved structural moieties that are found in microorganisms and are often called pathogen-associated molecular patterns (PAMPs) (for review see Chen and Nuñez, 2010). Following ligand recognition these receptors activate downstream signaling pathways, such as the nuclear factor-κb (NF-κb), mitogen-activated protein kinase (MAPK) and type I interferon pathways, which result in the upregulation of pro-inflammatory cytokines and chemokines that are important in inflammatory responses. In non-infectious conditions immune cells can be activated via recognition of endogenous material by PPRs. These endogenous molecules have been named danger-associated molecular patterns (DAMPs). Under physiological conditions these DAMPs are localized intracellularly. Under conditions of apoptotic cells death, cells are cleared immunologically silent without significant release of DAMPs into the extracellular environment. In contrast, necrosis following ischemia leads to loss of cell integrity and release of the cell content into the extracellular space. DAMPs derived from necrotic cells include the chromatin-associated protein high-mobility group box 1 (HMGB1), heat shock proteins (HSPs), mitochondrial peptides and purine metabolites, such as adenosine triphosphate (ATP) and uric acid (reviewed by Chen and Nuñez, 2010 and Shen et al., 2013). Consecutively, activated receptors and signaling pathways include TLR2/4/9, CD24, CD44, NLRP3, formyl peptide receptor 1, RAGE and IL-1 receptor. In the context of stroke DAMPs are massively released into the extracellular compartment. In stroke several pathways have been described, including TLR2/4, CD38, P2X7 and RAGE, which are associated with an worsened outcome (Liu et al., 2007; Tang et al., 2008; Choe et al., 2011; Arbeloa et al., 2012). As we discussed above γδ T cells can be activated directly by DAMPs via TLR1/2 and dectin receptors and cytokines, such as IL-1β and IL-23 (Martin et al., 2009; Sutton et al., 2012). Following stroke, there is clear evidence that IL-23 activates IL-17 production in γδ T cells (Shichita et al., 2009). Even though it is likely that further signals via TcR and TLR/dectin receptors are necessary to fully activate γδ T cells, the actual experimental data is outstanding.

## Effector mechanisms of γδ T cells in stroke

Several papers have shown a significant contribution of γδ T cells and IL-17 in stroke and other conditions of central nervous system inflammation (Kebir et al., 2007; Shichita et al., 2009; Caccamo et al., 2011; Gelderblom et al., 2012). In ischemia reperfusion injury of the brain we and others have observed a pathogenic role of γδ T cells, which can be detected in ischemic brain tissue as early as 6 h post ischemia (Shichita et al., 2009; Gelderblom et al., 2012). Effector mechanisms of γδ T cells in stroke primarily depend on their IL-17 production. In stroke, synergistic stimulation of astrocytes by IL-17 and TNF-α induces a massive induction of neutrophil attracting chemokines including CXCL-1 (Gelderblom et al., 2012), resulting in a subsequent neutrophil infiltration, which is leading to an increased tissue damage. Activated macrophages and microglia are secreting high amounts of TNF-α in the ischemic tissue. In the presence of the TNF-α rich milieu the additional IL-17 signal leads to the rapid increase of CXCL-1 via a stabilizing effect on the CXCL-1 RNA in astrocytes. Blocking either signal, IL-17 or the CXCL-1/CXCR2-axis, results in a robust reduction in infarct size and a significant improved neurological outcome. Even if an anti-IL-17 antibody is administered 6 h after stroke, neutrophil invasion can be blocked (Gelderblom et al., 2012). Neutrophil independent effects of IL-17 secreted by γδ T cells in stroke include the induction of MMP3 and MMP9 which are associated with blood brain barrier breakdown (Shichita et al., 2009; Gelderblom et al., 2012). Other potential effector functions of γδ T are engagement of death inducing receptors such as CD95 or TNF-related apoptosis-inducing ligand receptors (TRAILR), and the release of cytotoxic effector molecules, such as perforin and granzymes (See Figure 1). Molecular signals directing γδ T cell into the ischemic brain is another unresolved issue. γδ T cell subpopulations can be divided by Scart-2 and CCR6 vs. NK.1.1 and CD27 expression into IL-17 vs. IFN-γ producing T cells, respectively. The functional relevance of the CCR6 expression on IL-17 producing γδ T cells is supported by experimental data, showing that the migration of γδ T cells into the inflamed liver depends on the CCL20/CCR6 axis (Hammerich et al., 2014). Nevertheless, in stroke it is so far unclear which chemokines/chemokine receptors are essential for the entry of γδ T cells into the ischemic brain and which γδ T cell subpopulations are migrating into the ischemic brain.

## Role of γδ T cells in human stroke pathophysiology

Most of the data on inflammation in stroke is derived from studies in rodent models. These models have several drawback, including differences between the immune system of rodents and humans. Further, the vast majority of stroke patients are older that 65 and are characterized by co-morbidities, which are not reflected in rodent models (Heuschmann et al., 2010). Despite these discrepancies, results from post-mortem and imaging studies in human stroke demonstrate that a rapid activation of the resident and systemic immune system are hallmarks of human stroke pathophysiology (Mena et al., 2004; Price et al., 2004; Thiel and Heiss, 2011). Similar to experimental stroke, neutrophils are recruited into the ischemic brain within 24 h after symptom onset (Chuaqui and Tapia, 1993; Price et al., 2004) and microglia undergo rapid activation in the infarct core but also remote areas such as fiber tracts or relay nuclei (Thiel and Heiss, 2011). These findings let to several clinical trials targeting neutrophils in human stroke. Studies employing inhibitors of the neutrophil—endothelial cell interaction including CD18 and ICAM-1 were conducted, none of them showing favorable results on the clinical outcome parameters (del Zoppo, 2010). Nevertheless, the immunological understanding of the post ischemic inflammatory response was limited when these human trials were designed. Regarding our current understanding of the stroke induced inflammation IL-17 seems to be promising target. Infiltration by γδ T cells and secretion of IL-17 have been demonstrated in ischemic pathological human brain tissue (Li et al., 2005; Gelderblom et al., 2012). Similarly, IL-17 induced downstream pathways can be found. The IL-17 presence in the ischemic brain is early and short-lived and has most likely only pro-inflammatory effects. Therefore a short anti-IL-17 intervention could be beneficial without producing side effects, for example enhancing the systemic immune suppression. Recent data from human clinical trials with humanized neutralizing IL-17A antibodies in patients with autoimmune disease showed that treatment is well tolerized and effective (Hueber et al., 2010).

## Summary

Inflammation can enhance ischemic damage and lymphocytes seem to be important component of this process. Interestingly, the classical concepts of adaptive immune responses do not explain all observed effects. Several innate like features of lymphocytes dominate the early pro-inflammatory events. Particularly atypical T cells such as γδ T cells could explain some of these discrepancies and targeted treatment against their signature cytokine IL-17 might be a promising treatment option.

## Conflict of interest statement

The authors declare that the research was conducted in the absence of any commercial or financial relationships that could be construed as a potential conflict of interest.

## References

[B1] Arbeloa J. Pérez-Samartín A. Gottlieb M. Matute C. (2012). P2X7 receptor blockade prevents ATP excitotoxicity in neurons and reduces brain damage after ischemia. Neurobiol. Dis. 45, 954–961. 10.1016/j.nbd.2011.12.014 22186422

[B2] Becker K. Kindrick D. McCarron R. Hallenbeck J. Winn R. (2003). Adoptive transfer of myelin basic protein-tolerized splenocytes to naive animals reduces infarct size: a role for lymphocytes in ischemic brain injury? Stroke 34, 1809–1815. 10.1161/01.str.0000078308.77727.ea 12791945

[B3] Becker K. J. McCarron R. M. Ruetzler C. Laban O. Sternberg E. Flanders K. C.. (1997). Immunologic tolerance to myelin basic protein decreases stroke size after transient focal cerebral ischemia. Proc. Natl. Acad. Sci. U S A 94, 10873–10878. 10.1073/pnas.94.20.10873 9380727 PMC23514

[B4] Bonneville M. O’Brien R. L. Born W. K. (2010). Gammadelta T cell effector functions: a blend of innate programming and acquired plasticity. Nat. Rev. Immunol. 10, 467–478. 10.1038/nri2781 20539306

[B5] Caccamo N. La Mendola C. Orlando V. Meraviglia S. Todaro M. Stassi G.. (2011). Differentiation, phenotype and function of interleukin-17-producing human Vγ9Vδ2 T cells. Blood 118, 129–138. 10.1182/blood-2011-01-331298 21505189

[B6] Chen G. Y. Nuñez G. (2010). Sterile inflammation: sensing and reacting to damage. Nat. Rev. Immunol. 10, 826–837. 10.1038/nri2873 21088683 PMC3114424

[B7] Choe C.-U. Lardong K. Gelderblom M. Ludewig P. Leypoldt F. Koch-Nolte F.. (2011). CD38 exacerbates focal cytokine production, postischemic inflammation and brain injury after focal cerebral ischemia. PLoS One 6:e19046. 10.1371/journal.pone.0019046 21625615 PMC3097994

[B8] Chuaqui R. Tapia J. (1993). Histologic assessment of the age of recent brain infarcts in man. J. Neuropathol. Exp. Neurol. 52, 481–489. 10.1097/00005072-199309000-00006 8360701

[B9] Clarkson B. D. S. Ling C. Shi Y. Harris M. G. Rayasam A. Sun D.. (2014). T cell-derived interleukin (IL)-21 promotes brain injury following stroke in mice. J. Exp. Med. 211, 595–604. 10.1084/jem.20131377 24616379 PMC3978271

[B10] del Zoppo G. J. (2010). Acute anti-inflammatory approaches to ischemic stroke. Ann. N Y Acad. Sci. 1207, 143–148. 10.1111/j.1749-6632.2010.05761.x 20955437 PMC4552338

[B12] Frenkel D. Huang Z. Maron R. Koldzic D. N. Moskowitz M. A. Weiner H. L. (2005). Neuroprotection by IL-10-producing MOG CD4+ T cells following ischemic stroke. J. Neurol. Sci. 233, 125–132. 10.1016/j.jns.2005.03.022 15894335

[B13] Gan Y. Liu Q. Wu W. Yin J. X. Bai X. F. Shen R.. (2014). Ischemic neurons recruit natural killer cells that accelerate brain infarction. Proc. Natl. Acad. Sci. U S A 111, 2704–2709. 10.1073/pnas.1315943111 24550298 PMC3932858

[B14] Gelderblom M. Weymar A. Bernreuther C. Velden J. Arunachalam P. Steinbach K.. (2012). Neutralization of the IL-17 axis diminishes neutrophil invasion and protects from ischemic stroke. Blood 120, 3793–3802. 10.1182/blood-2012-02-412726 22976954

[B15] Hammerich L. Bangen J. M. Govaere O. Zimmermann H. W. Gassler N. Huss S.. (2014). Chemokine receptor CCR6-dependent accumulation of γδ T cells in injured liver restricts hepatic inflammation and fibrosis. Hepatology 59, 630–642. 10.1002/hep.26697 23959575 PMC4139146

[B16] Heuschmann P. Busse O. Wagner M. Endres M. Villringer A. Röther J. (2010). Schlaganfallhäufigkeit und Versorgung von Schlaganfallpatienten in Deutschland. Akt. Neurol. 37, 333–340 10.1055/s-0030-1248611

[B17] Hueber W. Patel D. D. Dryja T. Wright A. M. Koroleva I. Bruin G.. (2010). Effects of AIN457, a fully human antibody to Interleukin-17A, on Psoriasis, Rheumatoid Arthritis and Uveitis. Sci. Transl. Med. 2:52ra72. 10.1126/scitranslmed.3001107 20926833

[B18] Hurn P. D. Subramanian S. Parker S. M. Afentoulis M. E. Kaler L. J. Vandenbark A. A.. (2007). T- and B-cell-deficient mice with experimental stroke have reduced lesion size and inflammation. J. Cereb. Blood Flow Metab. 27, 1798–1805. 10.1038/sj.jcbfm.9600482 17392692 PMC2592689

[B19] Iadecola C. Anrather J. (2011). The immunology of stroke: from mechanisms to translation. Nat. Med. 17, 796–808. 10.1038/nm.2399 21738161 PMC3137275

[B20] Jensen K. D. C. Su X. Shin S. Li L. Youssef S. Yamasaki S.. (2008). Thymic selection determines γδ T cell effector fate: antigen-naive cells make interleukin-17 and antigen-experienced cells make interferon γ. Immunity 29, 90–100. 10.1016/j.immuni.2008.04.022 18585064 PMC2601709

[B21] Kebir H. Kreymborg K. Ifergan I. Dodelet-Devillers A. Cayrol R. Bernard M.. (2007). Human TH17 lymphocytes promote blood-brain barrier disruption and central nervous system inflammation. Nat. Med. 13, 1173–1175. 10.1038/nm1651 17828272 PMC5114125

[B22] Kleinschnitz C. Kraft P. Dreykluft A. Hagedorn I. Göbel K. Schuhmann M. K.. (2013). Regulatory T cells are strong promoters of acute ischemic stroke in mice by inducing dysfunction of the cerebral microvasculature. Blood 121, 679–691. 10.1182/blood-2012-04-426734 23160472 PMC3790947

[B23] Kleinschnitz C. Schwab N. Kraft P. Hagedorn I. Dreykluft A. Schwarz T.. (2010). Early detrimental T-cell effects in experimental cerebral ischemia are neither related to adaptive immunity nor thrombus formation. Blood 115, 3835–3842. 10.1182/blood-2009-10-249078 20215643

[B24] Korn T. Petermann F. (2012). Development and function of interleukin 17-producing γδ T cells. Ann. N Y Acad. Sci. 1247, 34–45. 10.1111/j.1749-6632.2011.06355.x 22239719

[B25] Li P. Gan Y. Sun B.-L. Zhang F. Lu B. Gao Y.. (2013). Adoptive regulatory T-cell therapy protects against cerebral ischemia. Ann. Neurol. 74, 458–471. 10.1002/ana.23815 23674483 PMC3748165

[B26] Li P. Mao L. Liu X. Gan Y. Zheng J. Thomson A. W.. (2014). Essential role of program death 1-Ligand 1 in regulatory T-cell-afforded protection against blood-brain barrier damage after stroke. Stroke 45, 857–864. 10.1161/STROKEAHA.113.004100 24496394 PMC3939692

[B27] Li G.-Z. Zhong D. Yang L.-M. Sun B. Zhongy Z.-H. Yin Y.-H.. (2005). Expression of interleukin-17 in ischemic brain tissue. Scand. J. Immunol. 62, 481–486. 10.1111/j.1365-3083.2005.01683.x 16305645

[B28] Liesz A. Suri-Payer E. Veltkamp C. Doerr H. Sommer C. Rivest S.. (2009). Regulatory T cells are key cerebroprotective immunomodulators in acute experimental stroke. Nat. Med. 15, 192–199. 10.1038/nm.1927 19169263

[B29] Liesz A. Zhou W. Mracskó E. Karcher S. Bauer H. Schwarting S.. (2011). Inhibition of lymphocyte trafficking shields the brain against deleterious neuroinflammation after stroke. Brain 134, 704–720. 10.1093/brain/awr008 21354973

[B30] Liesz A. Zhou W. Na S. Y. Hämmerling G. J. Garbi N. Karcher S.. (2013). Boosting regulatory T cells limits neuroinflammation in permanent cortical stroke. J. Neurosci. 33, 17350–17362. 10.1523/JNEUROSCI.4901-12.2013 24174668 PMC6618366

[B31] Liu K. Mori S. Takahashi H. K. Tomono Y. Wake H. Kanke T.. (2007). Anti-high mobility group box 1 monoclonal antibody ameliorates brain infarction induced by transient ischemia in rats. FASEB J. 21, 3904–3916. 10.1096/fj.07-8770com 17628015

[B32] Martin B. Hirota K. Cua D. J. Stockinger B. Veldhoen M. (2009). Interleukin-17-producing gammadelta T cells selectively expand in response to pathogen products and environmental signals. Immunity 31, 321–330. 10.1016/j.immuni.2009.06.020 19682928

[B33] Mena H. Cadavid D. Rushing E. J. (2004). Human cerebral infarct: a proposed histopathologic classification based on 137 cases. Acta Neuropathol. 108, 524–530. 10.1007/s00401-004-0918-z 15517310

[B34] Price C. J. S. Menon D. K. Peters A. M. Ballinger J. R. Barber R. W. Balan K. K.. (2004). Cerebral neutrophil recruitment, histology and outcome in acute ischemic stroke: an imaging-based study. Stroke 35, 1659–1664. 10.1161/01.str.0000130592.71028.92 15155970

[B35] Raulet D. H. (1989). The structure, function, and molecular genetics of the gamma/delta T cell receptor. Annu. Rev. Immunol. 7, 175–207. 10.1146/annurev.immunol.7.1.175 2653369

[B36] Ren X. Akiyoshi K. Vandenbark A. A. Hurn P. D. Offner H. (2011). CD4+FoxP3+ regulatory T-cells in cerebral ischemic stroke. Metab. Brain Dis. 26, 87–90. 10.1007/s11011-010-9226-6 21082336 PMC3070853

[B37] Roark C. L. French J. D. Taylor M. A. Bendele A. M. Born W. K. O’Brien R. L. (2007). Exacerbation of collagen-induced arthritis by oligoclonal, IL-17-producing γδ T cells. J. Immunol. 179, 5576–5583. 10.4049/jimmunol.179.8.5576 17911645 PMC2768546

[B38] Sharkey J. Butcher S. P. (1994). Immunophilins mediate the neuroprotective effects of Fk506 in focal cerebral-ischemia. Nature 371, 336–339. 10.1038/371336a0 7522303

[B39] Shen H. Kreisel D. Goldstein D. R. (2013). Processes of Sterile Inflammation. J. Immunol. 191, 2857–2863. 10.4049/jimmunol.1301539 24014880 PMC3787118

[B40] Shibata K. Yamada H. Hara H. Kishihara K. Yoshikai Y. (2007). Resident Vdelta1+ gammadelta T cells control early infiltration of neutrophils after Escherichia coli infection via IL-17 production. J. Immunol. 178, 4466–4472. 10.4049/jimmunol.178.7.4466 17372004

[B41] Shichita T. Sugiyama Y. Ooboshi H. Sugimori H. Nakagawa R. Takada I.. (2009). Pivotal role of cerebral interleukin-17-producing gammadeltaT cells in the delayed phase of ischemic brain injury. Nat. Med. 15, 946–950. 10.1038/nm.1999 19648929

[B42] Simonian P. L. Roark C. L. Wehrmann F. Lanham A. M. Born W. K. O’Brien R. L.. (2009). IL-17A-Expressing T Cells are essential for bacterial clearance in a Murine Model of Hypersensitivity Pneumonitis. J. Immunol. 182, 6540–6549. 10.4049/jimmunol.0900013 19414809 PMC2766088

[B43] Sutton C. E. Lalor S. J. Sweeney C. M. Brereton C. F. Lavelle E. C. Mills K. H. G. (2009). Interleukin-1 and IL-23 induce innate IL-17 production from gammadelta T cells, amplifying Th17 responses and autoimmunity. Immunity 31, 331–341. 10.1016/j.immuni.2009.08.001 19682929

[B44] Sutton C. E. Mielke L. A. Mills K. H. G. (2012). IL-17-producing γδ T cells and innate lymphoid cells. Eur. J. Immunol. 42, 2221–2231. 10.1002/eji.201242569 22949320

[B45] Tang S.-C. Lathia J. D. Selvaraj P. K. Jo D.-G. Mughal M. R. Cheng A.. (2008). Toll-like receptor-4 mediates neuronal apoptosis induced by amyloid beta-peptide and the membrane lipid peroxidation product 4-hydroxynonenal. Exp. Neurol. 213, 114–121. 10.1016/j.expneurol.2008.05.014 18586243 PMC2597513

[B46] Thiel A. Heiss W.-D. (2011). Imaging of microglia activation in stroke. Stroke 42, 507–512. 10.1161/STROKEAHA.110.598821 21164114

[B47] Vantourout P. Hayday A. (2013). Six-of-the-best: unique contributions of. Nat. Rev. Immunol. 13, 88–100. 10.1038/nri3384 23348415 PMC3951794

[B48] Yilmaz G. Arumugam T. V. Stokes K. Y. Granger D. N. (2006). Role of T lymphocytes and interferon-gamma in ischemic stroke. Circulation 113, 2105–2112. 10.1161/circulationaha.105.593046 16636173

[B49] Yilmaz G. Granger D. N. (2008). Cell adhesion molecules and ischemic stroke. Neurol. Res. 30, 783–793. 10.1179/174313208X341085 18826804 PMC2748428

